# Repurposing Pomalidomide as a Neuroprotective Drug: Efficacy in an Alpha-Synuclein-Based Model of Parkinson’s Disease

**DOI:** 10.1007/s13311-022-01182-2

**Published:** 2022-01-24

**Authors:** Maria Francesca Palmas, Anna Ena, Chiara Burgaletto, Maria Antonietta Casu, Giuseppina Cantarella, Ezio Carboni, Michela Etzi, Alfonso De Simone, Giuliana Fusco, Maria Cristina Cardia, Francesco Lai, Luca Picci, David Tweedie, Michael T. Scerba, Valentina Coroneo, Renato Bernardini, Nigel H. Greig, Augusta Pisanu, Anna R. Carta

**Affiliations:** 1 Department of Biomedical Sciences, University of Cagliari, Cagliari, Italy; 2 Department of Biomedical and Biotechnological Sciences, University of Catania, Catania, Italy; 3 CNR Institute of Translational Pharmacology, Cagliari, Italy; 4 Department of Pharmacy, University of Naples Federico II, Naples, Italy; 5 Centre for Misfolding Diseases, Department of Chemistry, University of Cambridge, Cambridge, UK; 6 Department of Life and Environmental Sciences, University of Cagliari, Cagliari, Italy; 7 Drug Design & Development Section, Translational Gerontology Branch, National Institute On Aging, National Institutes of Health, Baltimore, MD USA; 8 Department of Medical Sciences and Public Health, University of Cagliari, Cagliari, Italy; 9 National Research Council, Institute of Neuroscience, Cagliari, Italy

**Keywords:** Immunomodulation, Alpha-synuclein, Cytokine, Neuroprotection, Motor impairment, Drug repositioning

## Abstract

Marketed drugs for Parkinson’s disease (PD) treat disease motor symptoms but are ineffective in stopping or slowing disease progression. In the quest of novel pharmacological approaches that may target disease progression, drug-repurposing provides a strategy to accelerate the preclinical and clinical testing of drugs already approved for other medical indications. Here, we targeted the inflammatory component of PD pathology, by testing for the first time the disease-modifying properties of the immunomodulatory imide drug (IMiD) pomalidomide in a translational rat model of PD neuropathology based on the intranigral bilateral infusion of toxic preformed oligomers of human α-synuclein (H-αSynOs). The neuroprotective effect of pomalidomide (20 mg/kg; i.p. three times/week 48 h apart) was tested in the first stage of disease progression by means of a chronic two-month administration, starting 1 month after H-αSynOs infusion, when an already ongoing neuroinflammation is observed. The intracerebral infusion of H-αSynOs induced an impairment in motor and coordination performance that was fully rescued by pomalidomide, as assessed via a battery of motor tests three months after infusion. Moreover, H-αSynOs-infused rats displayed a 40–45% cell loss within the bilateral substantia nigra, as measured by stereological counting of TH + and Nissl-stained neurons, that was largely abolished by pomalidomide. The inflammatory response to H-αSynOs infusion and the pomalidomide treatment was evaluated both in CNS affected areas and peripherally in the serum. A reactive microgliosis, measured as the volume occupied by the microglial marker Iba-1, was present in the substantia nigra three months after H-αSynOs infusion as well as after H-αSynOs plus pomalidomide treatment. However, microglia differed for their phenotype among experimental groups. After H-αSynOs infusion, microglia displayed a proinflammatory profile, producing a large amount of the proinflammatory cytokine TNF-α. In contrast, pomalidomide inhibited the TNF-α overproduction and elevated the anti-inflammatory cytokine IL-10. Moreover, the H-αSynOs infusion induced a systemic inflammation with overproduction of serum proinflammatory cytokines and chemokines, that was largely mitigated by pomalidomide. Results provide evidence of the disease modifying potential of pomalidomide in a neuropathological rodent model of PD and support the repurposing of this drug for clinical testing in PD patients.

**Supplementary Information:**

The online version contains supplementary material available at 10.1007/s13311-022-01182-2.

## Introduction

Despite massive preclinical and clinical effort, marketed drugs for the treatment of PD remain symptomatic in their efficacy and appear ineffective in stopping or slowing disease progression. An increasing knowledge of PD neuropathology together with the wide availability of drugs with regulatory-approved safety data, endorses drug-repurposing as an appealing strategy to accelerate the preclinical and clinical testing of drugs already approved for other medical indications [1–4]. In this quest, drugs targeting components of the inflammatory response represent an attractive disease-modifying strategy, and several clinically available immunosuppressive and immunomodulatory drugs have been tested for their neuroprotective activity in preclinical models of PD [5]. Chronic neuroinflammation is a recognized neuropathological trait of PD, originating from dysfunctional glial cells in the brain, likely driven by pathological interactions with toxic α-synuclein (α-Syn) [6, 7]. Glial cells in PD lose their homeostatic and defense function in favor of a pathological gain of toxic functions. This results in an unremittent production of proinflammatory molecules, such as tumor necrosis factor (TNF)-α, within degenerating mesencephalic areas [8–12]. In addition, analyses of serum and peripheral organs of PD patients have revealed a dysregulated cytokine content [13, 14], suggesting that the inflammatory pathology can involve the whole organism [15].

An attractive class of drugs in this context are the immunomodulatory imide drugs (IMiDs), such as thalidomide and its analogs, that have shown beneficial effects on neurodegeneration in preclinical models of PD [1, 16, 17]. Compared to drugs with immunosuppressive activity, whose clinical translation is often hampered by toxicological and pharmacokinetic limitations, IMiDs offer several advantageous features that make them more suitable for treating chronic neurological disorders. Most importantly, both in silico and in vivo data have shown that IMiDs cross the blood–brain barrier (BBB) and readily enter the brain from plasma [18, 19] (Cardia et al. submitted). IMiDs display a potent anti-inflammatory effect, acting primarily through the inhibition of TNF-α production via post-translational mechanisms, and consequent dampening of the inflammatory cascade [20–24]. Among FDA-approved IMiDs, pomalidomide is a regulatory-approved drug used in the oncology area for the treatment of specific types of cancer such as relapsed/refractory multiple myeloma and Kaposi sarcoma. Pomalidomide is particularly attractive because it displays a TNF-α inhibitory action of up to 50,000-fold greater than the parent compound thalidomide [25, 26], and it has a favorable BBB permeability in rodents [27] (Cardia et al. submitted). In a recent study, we have reported the neuroprotective efficacy of pomalidomide in the *Drosophila* LRRK2^WD40^ genetic model of PD, where we have shown that pomalidomide rescued the motor activity and attenuated the dopaminergic neuron damage and degeneration [16]. Moreover, as compared to thalidomide and derivatives, pomalidomide displays less adverse effects related to neurotoxic activity [25, 28].

Here, we tested the disease-modifying properties of pomalidomide, including the central and peripheral drug effects on inflammatory markers, in the α-Syn-based rodent model of PD obtained by the central bilateral infusion of toxic oligomers of α-Syn (αSynOs) into the rat substantia nigra pars compacta (SNpc) [29, 30]. Despite an increasing literature suggesting a neuroprotective property of IMiDs in PD, most studies have been designed in toxin-based models, whereas data from the α-Syn-based translational model remain scarce or totally absent [17]. Yet, the central neuropathological role of α-Syn is widely acknowledged, and modelling the α-Syn neuropathology of PD offers a valuable tool to investigate novel neuroprotective therapies, when compared with classical toxin-based models, to further bridge the gap between preclinical and clinical studies [31, 32]. In this light, there is an urgent need for additional studies in these translational models of PD to better define the efficacy of IMiDs that may foster the translation to their clinical testing.

In order to comprehensively assess the disease-modifying properties of pomalidomide we chronically treated αSynOs-infused rats with the drug and evaluated their motor performance and coordination ability with a battery of highly sensitive tests. Thereafter, to assess the neuroprotective activity of pomalidomide we analyzed stereologically the number of tyrosine hydroxylase (TH)^+^ cells within the SNpc. The inflammatory response induced by αSynOs infusion and the pomalidomide activity toward this pathological trait, was appraised both in CNS affected areas by immunofluorescence analysis of the phenotype of microglia, defined as Iba-1^+^ (ionized calcium-binding adapter molecule 1) cells, and peripherally in the serum by multiplex ELISA for cytokines and chemokines.

## Materials and Methods

All procedures were performed in accord with the ARRIVE guidelines and in accordance with the guidelines and protocols approved by the European Community (2010/63UE L 276 20/10/2010). Experimental protocols were approved by the Italian Ministry of Health (authorization N 766/2020-PR). All efforts were made to minimize animal pain and discomfort and to reduce the number of experimental animals used.

### Production of Recombinant H-αSyn

Recombinant human αSyn (H-αSyn) was purified in *E. coli* using plasmid pT7-7 encoding for the protein as previously described [30]. The expression was induced with 1 mM IPTG at 37 °C for 4 h. The cell lysate was centrifuged at 22,000* g* (Beckman Coulter, Brea, USA) for 30 min, and the supernatant was then heated for 20 min at 70 °C. After centrifugation at 22,000* g*, two steps of precipitation and centrifugation were employed and, in particular, 10 mg·mL^−1^ streptomycin sulfate was added to the supernatant for DNA precipitation. Subsequently, 360 mg·mL^−1^ ammonium sulfate was added to the supernatant to precipitate the recombinant H-αSyn. The obtained pellet was resuspended in 25 mM Tris–HCl, pH 7.7 and, after dialysis against the same buffer, loaded onto an anion exchange column (26/10 Q sepharose high performance, GE Healthcare, Little Chalfont, UK) to be eluted with a 0–1 M NaCl step gradient. Further purification was achieved by applying size exclusion chromatography (Hiload 26/60 Superdex 75 preparation grade, GE Healthcare). The purity of the sample was analyzed by SDS-PAGE, and the protein concentration was determined from the absorbance at 275 nm using an extinction coefficient of 5600 M^−1^·cm^−1^.

### Purification of H-αsynO

Toxic oligomeric samples were prepared from purified recombinant H-αSyn as previously described [29, 30]. Lyophilized protein was resuspended in PBS buffer at a pH of 7.4 and a concentration of 12 mg·mL^−1^, then passed through a 0.22 μm cutoff filter before incubation at 37 °C for 24 h without agitation. Residual fibrillar species were removed by ultracentrifugation for 1 h at 288,000* g*, and excess of monomers were removed using several filtration steps with 100 kDa cutoff membranes. Samples of the toxic H-αSyn oligomers prepared in this manner are stable for many days at room temperature, but in this study were used within 2 days of their production. At the end of the purification procedure, and prior to intracerebral inoculation, oligomers were tested for endotoxin contamination via the LAL (Limulus Amebocyte Lysate) assay (Kairosafe, Italy).

### Animals, Stereotaxic Surgery, and Pharmacological Treatment

Male Sprague–Dawley rats (275–300 g, Envigo) were housed in groups of three to four in standard conditions of temperature (21 ± 1 °C) and humidity (60%) under a 12 h light/dark cycle (lights on 7:00 A.M) with standard chow and water available ad libitum.

**Fig. 1:**
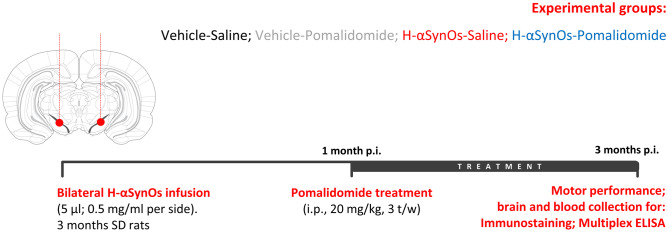
Experimental timeline showing the site for H-αSynOs infusion in vivo and the experimental protocol

Forty-five rats were deeply anesthetized with Fentanyl (3 mg/kg) and medetomidine hydrochloride (0.35 mg/kg) and were stereotaxically injected with 5 µL of human αSynO (H-αSynOs) into the SNpc (coordinates relative to bregma; −5.4 mm anteroposterior; ± 1.9 mm from the midline; −7.2 mm beneath the dura) bilaterally at the rate of 1 µL/min via a silica microinjector and according to the atlas of Paxinos and Watson [33], as previously described [29]. Control animals received sterile PBS (Fig. 1).

One-month post-surgery rats were chronically administered with pomalidomide (Pom, 20 mg/kg; i.p.) or saline on alternate days 3 days/week for 2 months and sacrificed 24 h after the last injection. The 1-month interval after infusion was chosen based on a previous observation [29] of neuronal damage and brain inflammation 30 days after oligomer infusion, which can be considered as a prodromal stage of PD, evolving into neuronal loss at three months post-infusion. Pomalidomide was nanosuspended in an aqueous solution of Tween 80 (0.75%) using the Wet media milling technique, as described [34] (Cardia et al. submitted), in order to optimize its bioavailability after i.p. injection. The experimental groups were as follows: (i) vehicle + saline (Veh-Sal); (ii) H-αSynOs + saline (H-αSynOs-Sal); (iii) Veh + Pom (Veh-Pom); (iv) H-αSynOs + Pom (H-αSynOs-Pom).

### Behavioral Tests

A range of behavioral studies were conducted over a 10-day interval, beginning 12 weeks after H-αSynOs infusion. Prior to each test, rats were acclimated to the testing room for 30 min in order to avoid any alteration in behavioral parameters induced by the novel environment. Tests were carried out between 9 am and 3 pm. All tests were performed and analyzed by individuals blinded to the experimental conditions.

### Challenging Beam Walk Test

The challenging beam test was used to assess motor coordination and balance as shown previously [29] with a protocol adapted from Drucker-Colín and García-Hernández [35], Fleming et al. [36], and Korecka et al. [37]. The testing apparatus consisted of a 2 m wooden beam placed between a starting platform, elevated 40 cm from the floor, and the home cage, with a slope of 15°. Three different beam widths were used: 15, 10, and 5 mm. All rats were trained to run across the different beams for 3 days before initiation of testing and, on the test day, they were videotaped. Briefly, each rat was placed at the lower end of the beam and the number of stepping errors was counted while traversing the beam to reach the home cage. The same procedure was repeated for the three different beam widths [29]. Some animal failed to complete the task within the established time-frame or fell off the beam, reflecting a sensorimotor impairment as previously reported [35]. In these events, in order to assign a numeric score to the impairment the error score was increased by adding a numerical increment based on the following criteria: (i) 0.25 increment, when the animal completed 75% of the beam; (ii) 0.5 increment, when the animal completed 50% of the beam; (iii) 0.75 when it only completed 25% of the beam.

### Vermicelli Handling Test

The vermicelli handling test was used as a measure of forepaw ability [38, 39]. During the week prior to testing, all rats were exposed to the pasta pieces several times in order to avoid any neophobic responses. On the test day, each rat was placed in a box with dark walls, with a mirror set 45° below it, and exposed to three 7 cm uncooked vermicelli strands. Each trial was videotaped for later analysis. Trials were invalidated should the rat break the pasta piece during eating or if less than 90% of the recorded eating session showed a clear view of the paws/digits [39, 40]. The primary outcome measures for this test were as follows: (a) number of forepaw adjustments per trial, defined as any distinct removal and replacement of the paw on the pasta piece; (b) frequency of atypical handling patterns. These atypical behaviors included the following: (1) paws together when long—paws placed symmetrically when the piece of pasta was 3.5 cm or greater in length; (2) guide and grasp switch—the roles of the guide limb and grasp limb are switched during eating; (3) failure to contact—the paw does not contact the pasta piece during eating; (4) drop—the pasta piece is dropped after eating is initiated; (5) paws apart when short—paws placed asymmetrically when the pasta piece is short [38]. These measures were scored 1 if exhibited and 0 when not, and their frequency was then summed.

### Gait Test

A gait test was run on an apparatus consisting of an arena placed on a base with a transparent floor and a mirror mounted inside. The arena was 15 cm wide, 25 cm tall, and 148 cm long. Walls and lid were made of black alveolar polypropylene panels to provide high contrast for white rodents. The base was a wooden frame (24 × 24 × 160 cm) provided with a plexiglass top, which served as a floor for the overlying arena. A mirror was housed inside and set 45° below the arena. Animals were acclimated to the arena for several minutes before testing. For testing, each rat independently and voluntarily explored the arena at self-selected velocities, and three runs across the arena were videotaped at 120 fps. For analysis, only the central portion of the arena was considered (100 cm long), whereas the starting and the terminal portions (24 cm each) were not considered. The time spent and the number of steps made to cross the central part of the arena were measured for each rat. Moreover, strides were analyzed by measuring the distance between paw placements for every step, on a stack of calibrated frames on IMAGE J (https://imagej.nih.gov/ij/). For each, subject data are expressed as averaged velocity (cm/s), steps per time unit (n/), and averaged stride length (cm).

### Immunohistochemistry

After the behavioral testing and within 24 h of their last injection, rats were anesthetized and transcardially perfused with ice-cold 0.1 M PBS (pH 7.4) followed by 4% buffered paraformaldehyde. After perfusion, the brain was carefully removed, post-fixed overnight in 4% paraformaldehyde-PBS and stored in 0.1% NaN_3_-PBS at 4 °C. Thereafter, 40-µm-thick serial sections of midbrain and striatum were vibratome-cut [41].

For stereological quantification, midbrain sections were pre-incubated in normal donkey serum and then immunoreacted with polyclonal rabbit anti-TH (1:1000, Millipore, Burlington, MA, USA) primary antibody. The reaction was amplified using a biotinylated secondary antibody and visualized by the classic avidin-peroxidase complex (ABC, Vector, UK) protocol, using 3,30-diaminobenzidine (Sigma-Aldrich, St. Louis, MO, USA) as a chromogen. Sections were then counterstained with cresyl violet.

For immunofluorescence, midbrain sections were pre-incubated with a blocking solution with normal serum/BSA and were then immunoreacted with the following unconjugated primary antibodies for double immunolabeling: goat polyclonal anti Iba-1 (1:1000; Novus Biologicals, Littleton, CO, USA); rabbit polyclonal anti TNF-α (1:500, Novus Biologicals, Littleton, CO, USA); rabbit polyclonal anti IL-10 (1:200, Abbiotec, Escondido, CA, USA); mouse monoclonal anti CD3 (1:50, Santa Cruz Biotechnology, Santa Cruz, CA, USA). For fluorescence visualization of Iba-1 and CD3 a two-step indirect labelling protocol was used, while a three-step detection was performed to increase the signal of TNF-α and IL-10 by combining biotin-SP-conjugated IgG (1:500, Jackson Immunoresearch, West Grove, PA, USA) and streptavidin–fluorescein (1:400, Jackson Immunoresearch, West Grove, PA, USA), as previously described [29]. Images were acquired using a spinning disk confocal microscope (Crisel Instruments, Rome, Italy) with a × 63 magnification.

### Stereological Counting of TH Immunoreactivity

All immunohistochemical reactions were analyzed by an operator blinded to the experimental groups, and different from the experimenter who performed the behavioral tests and histology. TH-immunoreactive neurons or Nissl-stained cells were counted bilaterally in the SNpc, as previously described [42]. A dedicated software was used (Stereologer, System Planning and Analysis, Inc., Alexandria, VA, USA), linked to a motorized stage on a BX-60 Olympus light microscope (Olympus, Segrate, Italy). The total number of TH-stained cells was estimated by means of the optical fractionator method, which combines the optical dissector with the fractionator sampling scheme, giving a direct estimation of the number of 3-D objects unbiased by shape, size, and orientation [43]. A systematic random sampling of cells within the area of interest was achieved by “Stereologer” software. Equidistant counting frames (frame area = 50 μm^2^) were obtained. Sampling fraction was delimited at low power and cells were sampled with a × 40 oil immersion objective through a defined depth with a 2 μm guard zone. The coefficient of error (CE) for each estimation and animal ranged from 0.05 to 0.1.

### Microscopy Analysis

Qualitative and quantitative analyses for Iba-1, TNF-α, IL-10, and CD3^+^ were performed using a spinning disk confocal microscope (Crisel Instruments, Rome, Italy) with a × 63 magnification. Surface rendering, colocalization, maximum intensity, and simulated fluorescence process algorithms were used (ImageJ and Imaris 7.3). To determine the Iba-1 occupied volume, a stack was obtained from each dataset (40 images). In the resulting stacks, 10 regions of interest for the SNpc (*x* = 700 μm; *y* = 700 μm; *z* = 40 μm) in each acquired section and for each animal were randomly chosen, and the volume of the elements calculated. For colocalization analysis, a colocalization channel was automatically generated by Imaris 7.3. In the resulting stacks each Iba-1^+^ cell was identified and selected, and the volume of the colocalized cytokine was calculated. Mean colocalization values obtained from cells analyzed in each animal from each experimental group (n = 6) were plotted as a frequency distribution displaying the percentage of colocalization between the selected cytokine signal and the selected Iba-1^+^ microglial cell. Histogram inspection indicated a different cytokine expression across experimental groups in a cell subpopulation, whereas a large population of cells maintained colocalization values similar to the vehicle (Fig. 6). For this reason, a deconvolution analysis was applied to the histograms in order to unmask subpopulations of cells affected by H-αSynOs infusion and/or pomalidomide treatment. Based on the deconvolution results, an appropriate cut-off value was set in order to categorize the identified cell populations into high and low expressing cells; mean values within each class were calculated for each experimental group and statistically compared.

### Cytokine and Chemokine Analysis by Multiplex ELISA

Serum samples were assayed using the Cytokine & Chemokine 22‐Plex Rat ProcartaPlexTM Panel (EPX220‐30,122‐901, Thermo Fisher Scientific, Vienna, Austria), according to the manufacturer’s instructions. The concentrations of cytokines and chemokines were detected with the Luminex MAGPIX instrument (Luminex Corporation, Austin, TX) and data were analyzed with xPONENT® software (Luminex Corporation, Austin, TX). Any analyte with a concentration outside the linear range was excluded from the analysis.

### Statistical Analysis

Outcome measures were evaluated by observers blinded to experimental conditions. Results are presented as mean ± SEM, using Statistica 8 (Stat Soft Inc., Tulsa, OK, USA). Behavioral data were analyzed by two-way analysis of variance (ANOVA) with intranigral infusion and pharmacological treatment as factors, followed by Tukey’s post hoc test, or by *t*-test where appropriate.

The results from the stereological analysis were statistically analyzed with a two-way analysis of variance (ANOVA) followed by Tukey’s post hoc test, whereas the dataset of Iba-1 IR in the SNpc was analyzed by a Kruskall-Wallis non-parametric test followed by Dunn’s post hoc test.

Regarding the colocalization analysis of the cytokines, the effect of drug treatments on the identified subpopulation was determined by one-way ANOVA followed by Fisher’s post hoc test for comparison between individual groups. Levels of serum cytokines and chemokines were statistically compared among the experimental groups by one-way ANOVA followed by Fisher’s post hoc test.

For all the analyses, the level of significance was set at *p* < 0.05.

## Results

### Pomalidomide Mitigates H-αSynOs Infusion-Induced Deficits in Sensorimotor Function and Fine Motor Movement Execution

**Fig. 2:**
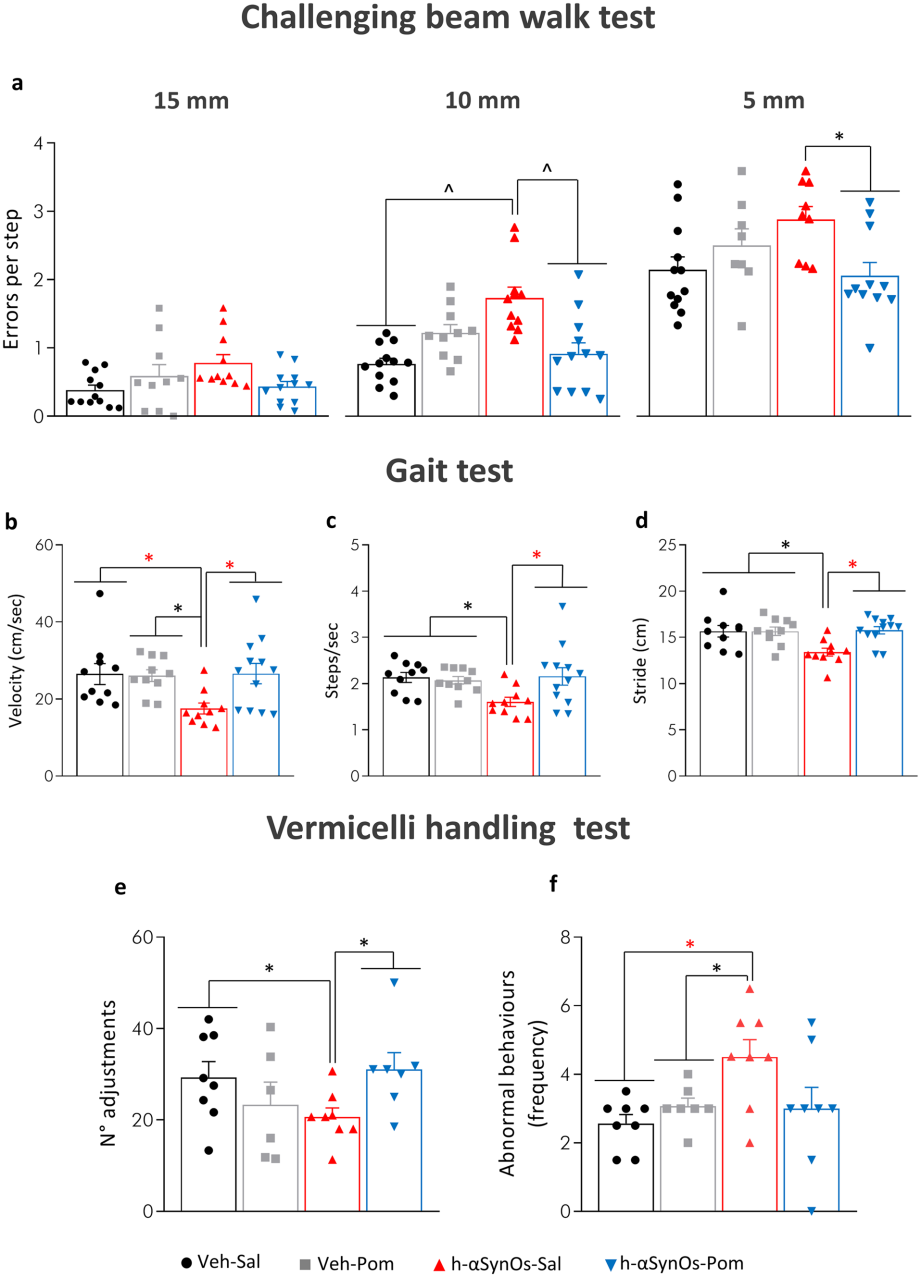
Chronic treatment with pomalidomide significantly reduced motor impairments 3 months after H-αSynOs infusion. Sensorimotor deficits were evaluated by the challenging beam walk test (**a**) and the gait test (**b–d**). Values represent the mean ± SEM (two-way ANOVA and Tukey’s post hoc test). ^*p* < 0.001; *p < 0.01; *p < 0.01. Fine motor movements were assessed by the vermicelli handling test. Stacked bar charts show the number of normal adjustments (**e**) and the frequency of abnormal behaviors (**f**) made during the eating time. Values represent the mean ± SEM. *p < 0.05 vs Veh-Sal and H-αSynOs-Pom (**e**); *p < 0.01 vs Veh-Sal (**f**) by *t*-test

To verify the functional outcome of pomalidomide-induced neuroprotection and specifically its efficacy against motor symptoms, a series of motor tests, including the challenging beam test, the vermicelli handling test and the gait test, were performed 3 months after H-αSynOs infusion. In line with our previous study [29], H-αSynOs-infused rats developed a significant motor impairment in the beam walking test, expressed as the number of errors per step on the 10 mm and 5 mm beams (Fig. 2a, a1, a2), which was significantly higher than in control rats (Veh-Sal group). Moreover, H-αSynOs-infused rats displayed an impairment in the gait test, expressed as a decrease of velocity, steps/second and stride length (Fig. 2b, b1, b2). Remarkably, pomalidomide treatment fully mitigated these sensorimotor impairments. In the beam test, pomalidomide-treated rats committed a similar number of stepping errors as the control group, which was significantly lower than H-αSynOs-infused rats for the 10 and 5 mm width beams (Fig. 2a). Likewise, in the gait test, pomalidomide treatment significantly restored velocity, number of steps/second and stride length, as compared to H-αSynOs-infused rats, to control values (Fig. 2b, c, d).

In the vermicelli handling test, H-αSynOs-infused rats showed fewer normal adjustments and an increased number of abnormal eating behaviors as compared with the Veh-Sal group (Fig. 2e, f), consistent with an impairment in fine movement execution. The administration of pomalidomide (H-αSynOs-Pom group) significantly increased the number of total adjustments during each trial, as compared to H-αSynOs-infused rats, and decreased the number of abnormal eating behaviors, restoring these to control values and indicating a recovery from this motor impairment (Fig. 2e, f).

### Pomalidomide Protects Against Nigral Dopaminergic Degeneration in H-αSynOs-Infused Rats

**Fig. 3:**
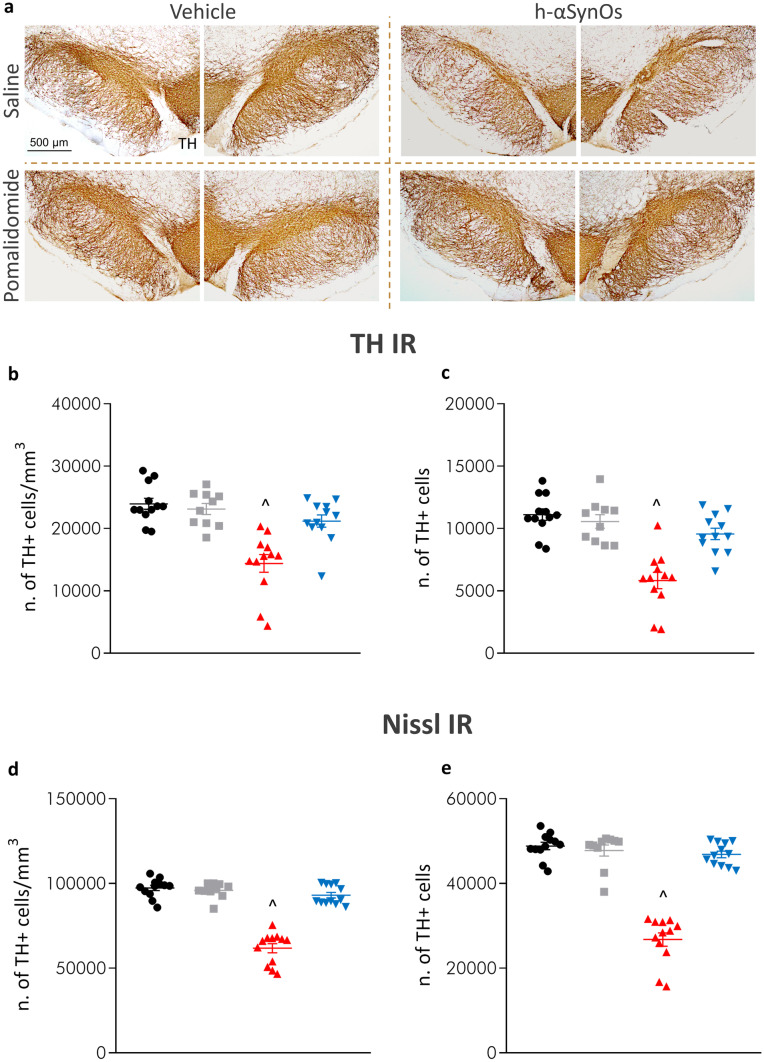
H-αSynOs induced a progressive nigrostriatal degeneration, which is rescued by pomalidomide treatment. **a** Representative images of TH-stained SNpc slices (magnification 5 ×), scale bar: 500 μm. **b** Density (expressed as number of TH^+^ cells/mm^3^) and **c** number of TH^+^ cells measured by stereological counting 3 months after the H-αSynOs or vehicle infusion and after 2 months treatment with pomalidomide or Saline. (**c-d**) Stereological quantification of Nissl-stained cells in the SNpc. Values represent the mean ± SEM. ^*p* < 0.001 vs all other groups, by two-way ANOVA and Tukey’s post hoc test

H-αSynOs-infused rats displayed a 40–45% reduction, in comparison with control rats, both in the density and the number of DA cells bilaterally in the right and left SNpc, as measured by stereological counting of TH^+^ neurons (Fig. 3a–c). Remarkably, such a reduction in the number of TH^+^ neurons was largely abolished when H-αSynOs-infused rats were chronically treated for 2 months with pomalidomide, starting one month post-oligomer infusion (Fig. 3a–c), indicating that pomalidomide stopped the neurodegenerative process. To confirm that the changes observed in TH content reflected changes in cell number rather than a decline/increase in enzyme levels, we stereologically counted Nissl-counterstained sections. Results were superimposable with our TH analysis, both in terms of density and number of cells, confirming that pomalidomide allayed H-αSynOs-induced cell loss in the SNpc (Fig. 3d, e).

### Pomalidomide Effects on H-αSynOs-Induced Microgliosis in the SNpc

**Fig. 4:**
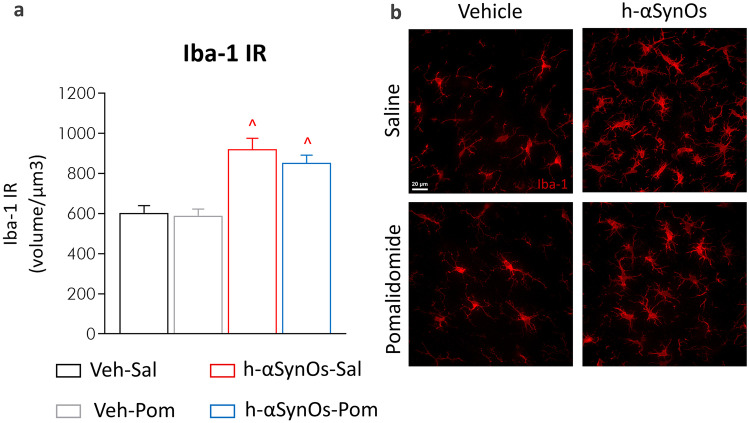
Microgliosis after H-αSynOs infusion with and without pomalidomide treatment. **a** Stacked bar chart shows the total volume occupied by Iba-1^+^ cells in the SNpc. **b** Representative images of Iba-1 cells (red, magnification 63X), scale bar: 20 μm. Values represent the mean ± SEM. ^*p* < 0.0001 vs. Veh-Sal and Veh-Pom, by Kruskall-Wallis followed by Dunn’s post hoc test

Based on the pivotal role of neuroinflammation in PD neuropathology [8] and the expected primary mechanism of pomalidomide activity, we evaluated microglial reactivity within the SNpc by using Iba-1 as a marker of reactive microglia. Results, expressed as the total volume occupied by Iba-1^+^ cells, are shown in Fig. 4a, b. Iba-1^+^ cell volume was similarly increased in the SNpc of H-αSynOs-infused rats treated with either saline or pomalidomide, suggesting that the pharmacological treatment did not reduce the microgliosis.

### Pomalidomide Switches the Functional Phenotype of Microglia in the H-αsynOs-Infused Rats

**Fig. 5:**
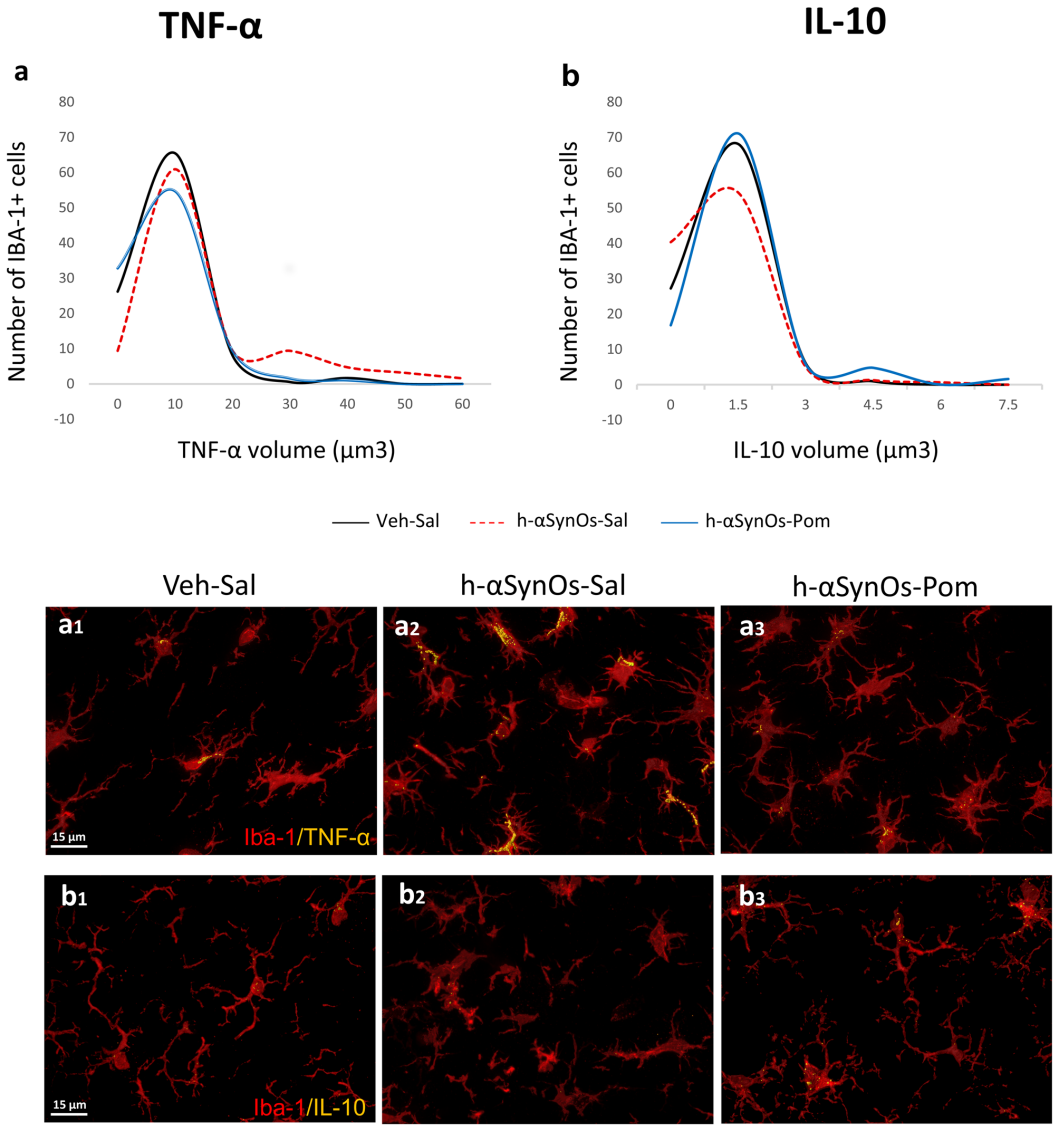
Pomalidomide reverses the imbalance of cytokines expression in the SNpc of H-αSynOs-infused rats. Frequency distribution of TNF-α (**a**) and IL-10 (**b**) colocalization within Iba-1 IR cells. Representative images of TNF-α (yellow) (a_1_–a_3_) and IL-10 (b_1_–b_3_) (yellow) colocalized with Iba-1^+^ cells (red). Magnification 63 × , scale bar: 15 μm

**Fig. 6:**
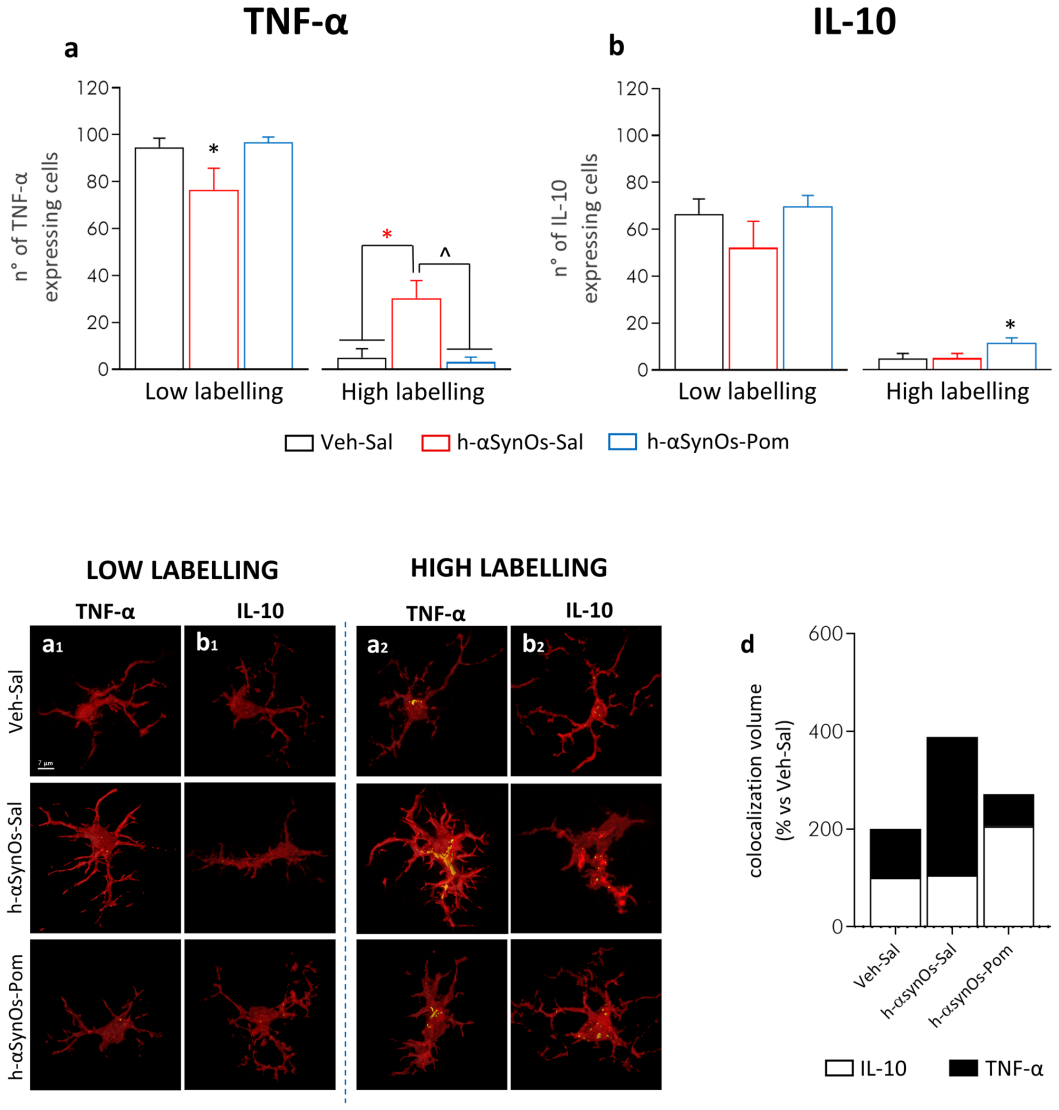
The functional phenotype of Iba-1^+^ microglia is altered by H-αSynOs infusion and pomalidomide treatment mitigates this. Iba-1^+^ cells were categorized into two sub-populations, based on the (**a**) TNF-α and (**b**) IL-10 expression, namely low labelled and high labelled. Values represent the mean ± SEM (one-way ANOVA followed by Tukey’s post hoc test). *p < 0.05; *p < 0.01; ^*p* < 0.001. **c** Representative images of TNF-α (yellow) and IL-10 low (c) and high (c_1_) expressing Iba-1^+^ cells (red). Magnification 63 × , scale bar: 7 μm. **d** Bar plot showing the relative percentage of each cytokine among the experimental groups

To further investigate the immunomodulatory activity of pomalidomide within the CNS, and find a mechanistic correlate of neuroprotection, we characterized the functional phenotype of Iba-1^+^ cells by cytokine assessment. A colocalization analysis was performed for the pro-inflammatory cytokine TNF-α and the anti-inflammatory cytokine IL-10 within microglial cells. Our analysis of the data revealed a high variability in the content of both TNF-α and IL-10 within microglia in each experimental group, as shown by the non-normal distribution of the dataset, reflecting the high functional dynamicity of these cells. Therefore, to highlight this aspect and to investigate how the αSynOs challenge and pomalidomide treatment affected the heterogeneity of cytokine content, data were expressed as a frequency distribution of cytokine volume within microglial cells (Fig. 5a, b). Whereas the histogram of the Veh-Sal group was characterized by a single peak/population that expressed low level of TNF-α, the histogram of the H-αSynOs-Sal group showed two peaks, produced by the low TNF-α expressing cells and a new—high TNF-α expressing population. Remarkably, this second population was abolished in the H-αSynOs-Pom group (Fig. 5a, a_1_-a_3_). These two populations of cells—low and high TNF-α labelled—that emerged from the deconvolution analysis, were separated by setting an appropriate cutoff value and statistically compared (Fig. 6a, b and corresponding images a_1_, a_2_, b_1_-b_2_). As shown in Fig. 6a, a significant difference was found among groups in the TNF-α content, both in the low and in the high labelled cells. Specifically, H-αSynOs-Sal rats showed a significant decrease of low labelled cells and an increase of highly labelled cells, as compared to control rats, suggesting that a subpopulation of microglia produced a supraphysiological amount of TNF-α (Fig. 6a, a_1_, a_2_). In contrast, H-αSynOs-Pom-treated rats displayed similar labelling to the control group for both cell populations, thereby indicating that pomalidomide treatment fully counteracted the H-αSynOs-induced increase above physiological values of the proinflammatory cytokine (Fig. 6a, a_1_, a_2_).

Microglia were further characterized by assessing the content of the anti-inflammatory cytokine IL-10 across experimental groups (Fig. 5b, b_1_-b_3_). As shown in Fig. 5b, histograms of all experimental groups were characterized by a large peak of cells showing low IL-10 labelling. However, the H-αSynOs-Sal histogram displayed a slight left-shift, which indicates a decrease in cytokine content. Moreover, the H-αSynOs-Pom histogram showed an additional small peak associated with higher IL-10 volumes. A statistical comparison of low and high labelled cells across experimental groups determined that the overall content of IL-10 was not affected by the H-αSynOs infusion; however, pomalidomide significantly increased the IL-10 content in a subpopulation of microglia above control values (Fig. 6b, b_1_, b_2_).

The percentage comparison of pro- versus anti-inflammatory microglia within the entire population analyzed, demonstrated that the H-αSynOs infusion induced a dysregulated ratio towards the pro-inflammatory form, whereas pomalidomide corrected such imbalance by inhibiting TNF-α and boosting IL-10 production (Fig. 6d). Collectively, our results show that pomalidomide, while not abolishing microgliosis and microglia reactivity, affected the microglia phenotype in an allostatic manner, to restore an optimal pro-/anti-inflammatory balance.

### Pomalidomide Mitigates H-αSynOs-Induced Systemic Inflammation

**Fig. 7:**
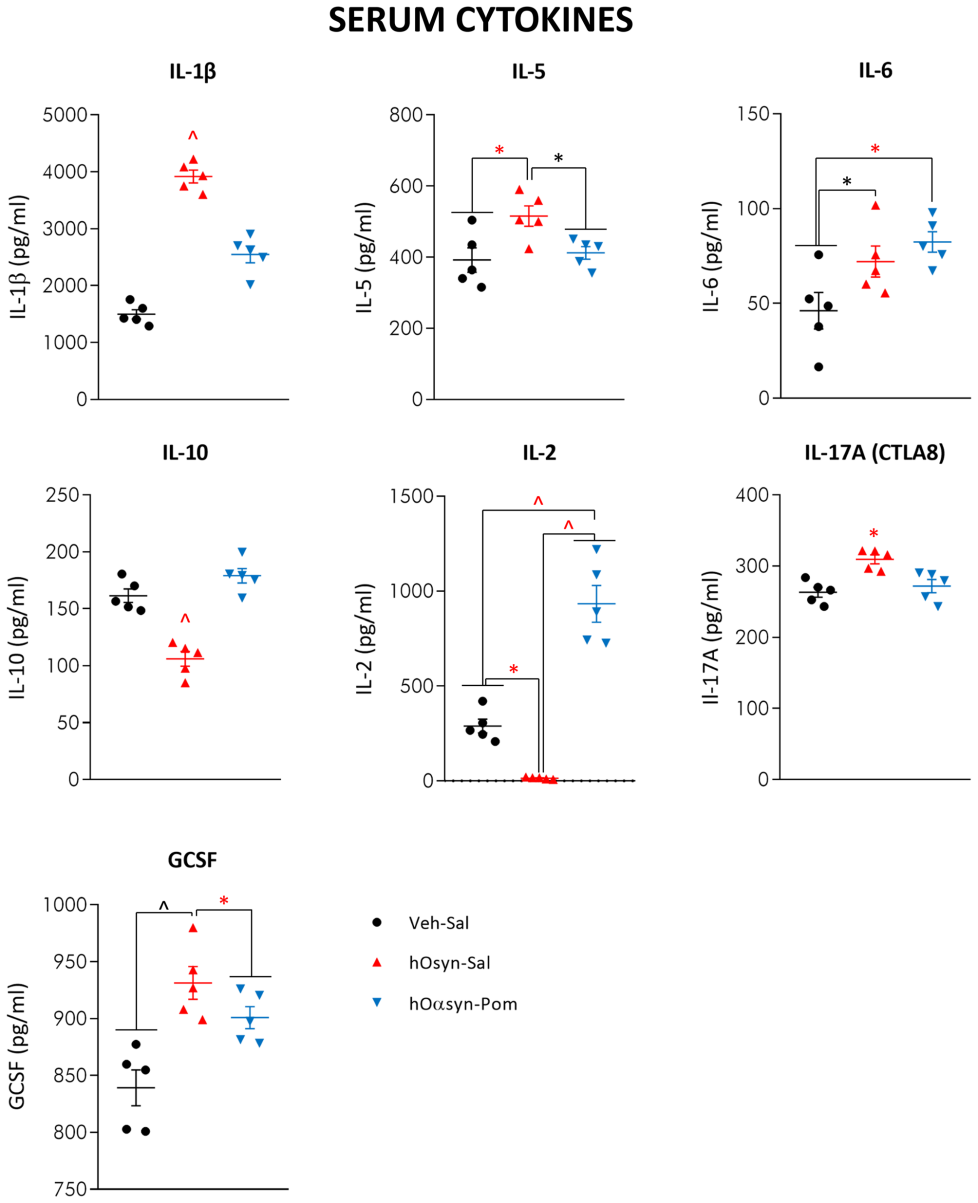
Serum cytokine content after H-αSynOs infusion and pomalidomide treatment. Serum cytokines were analyzed by multiplex ELISA. Values represent the mean ± SEM. ^*p* < 0.0001; ^*p* < 0.001; *p < 0.01; *p < 0.05, by one-way ANOVA and Fisher’s post hoc test

**Fig. 8:**
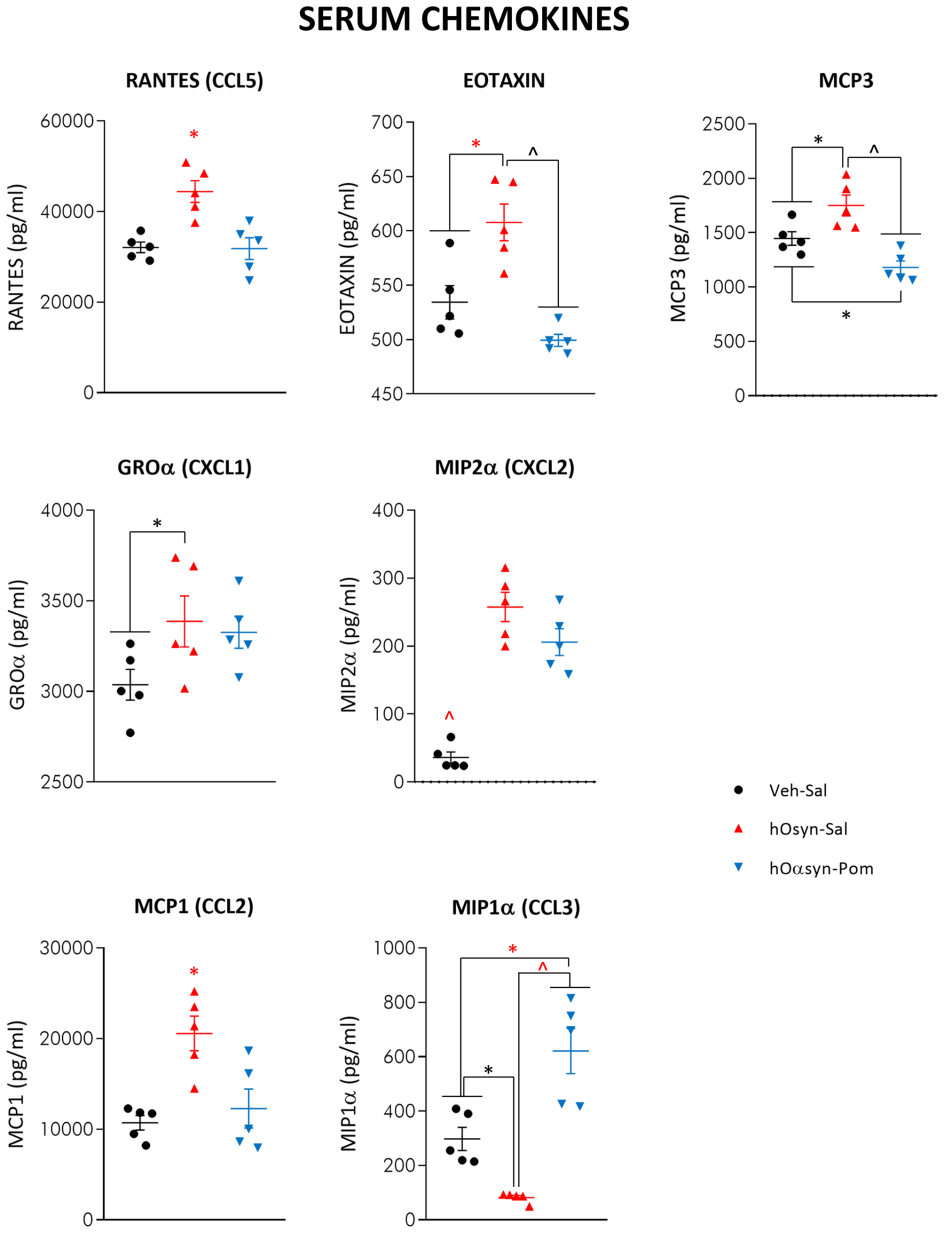
Serum chemokine content after H-αSynOs infusion and pomalidomide treatment. Serum chemokines were analyzed by multiplex ELISA. Values are the mean ± SEM. ^*p* < 0.0001; ^*p* < 0.001; *p < 0.01; *p < 0.05, by one-way ANOVA followed by Fisher’s post hoc test

**Fig. 9:**
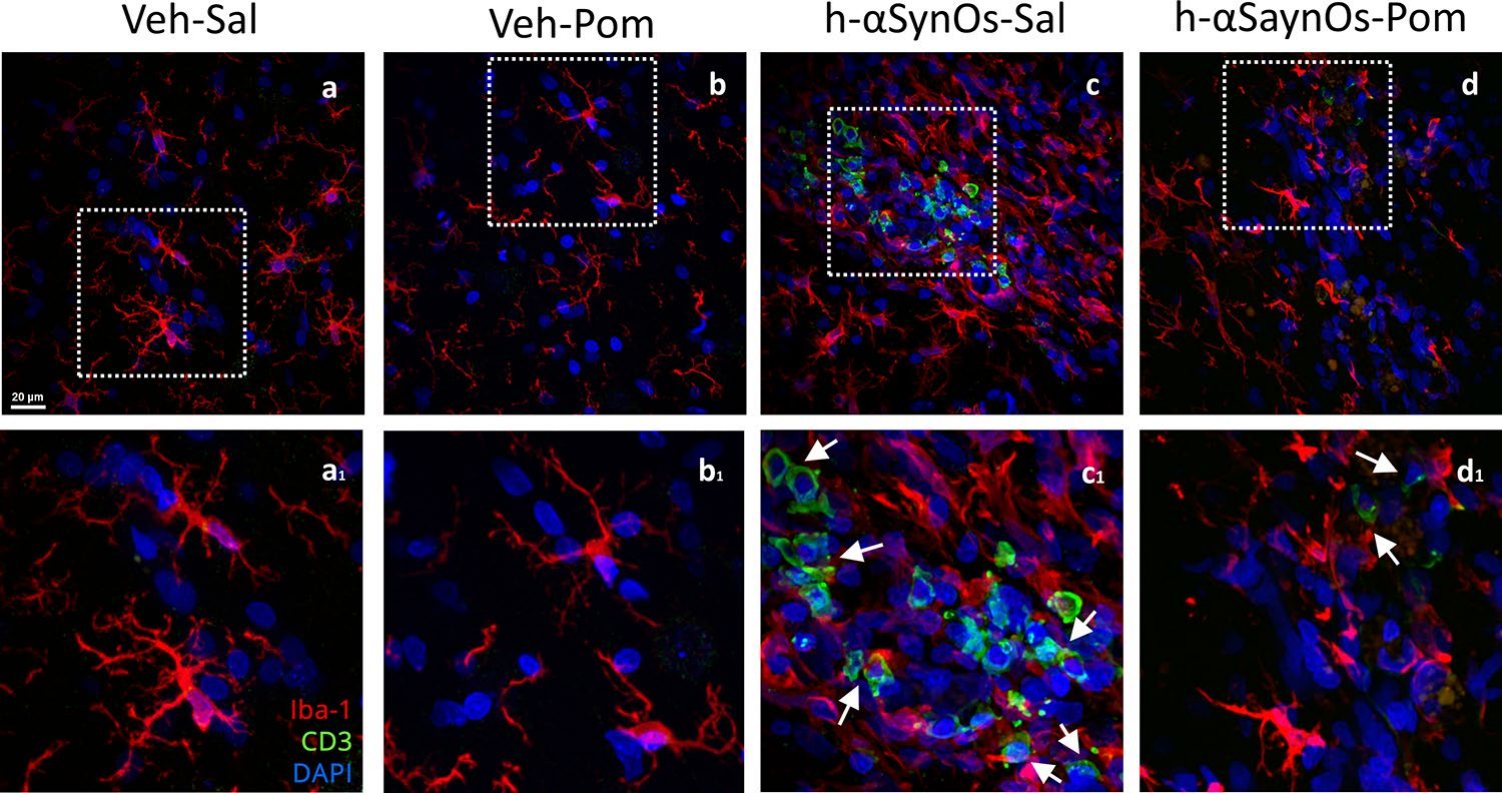
Representative pictures of the SNpc showing CD3^+^ T cells infiltration after H-αSynOs infusion and after pomalidomide treatment (a–d_1_) Double-immunostaining was performed using anti-CD3 (green) and anti-Iba-1 (red) antibodies and DAPI staining (blue, nuclei). Magnification 63 × , scale bar: 20 μm (**a**–**d**) and 10 μm (a_1_–d_1_)

The intracerebral infusion of H-αSynOs induced a systemic inflammation, as indicated by a profound dysregulation in serum levels of a panel of serum immune/inflammatory mediators, including cytokines and chemokines (Figs. 7 and 8). Specifically, serum levels of several pro-inflammatory molecules were elevated three months following the H-αSynOs intracerebral infusion, including cytokines IL-1β, IL-5, IL-6, IL-17, GCSF (Fig. 7), and chemokines RANTES, eotaxin, MCP3, CXCL 1/2, MCP1 (Fig. 8). Likewise notable, serum levels of the anti-inflammatory cytokine IL-10 as well as of IL-2 were decreased. Interestingly, serum TNF-α levels were not significantly affected by the infusion of αSynOs when comparing the serum of vehicle-infused rats (295.65 pg/mL) vs. H-αSynOs-infused rats (355.98 pg/mL), suggesting a different regulation of this cytokine at the central and peripheral level. In line with these increases in several chemokines, we observed an increase in CD3 immunostaining in the SNpc of H-αSynOs-infused rats, which did not colocalize with Iba-1, suggesting peripheral T-cell infiltration into the brain parenchyma (Fig. 9). Notably, pomalidomide reversed most of the αSynOs-induced changes in the serum, by restoring the physiological levels of several inflammatory and anti-inflammatory cytokines and chemokines, in line with the systemic immunomodulatory activity of this drug (Figs. 7 and 8). Accordingly, CD3 immunostaining was very low or absent in pomalidomide-treated rats (Fig. 9).

## Discussion

In the present study, we took advantage of a recently validated mammalian model of PD based on the intranigral infusion of toxic H-αSynOs [29] to evaluate the disease-modifying properties of the neuromodulatory IMiD drug pomalidomide. We found that pomalidomide was effective in mitigating the PD-related motor deficits and prevented the nigral dopaminergic cell loss induced by the intracerebral infusion of αSynOs. This effect was achieved by correcting the pathological phenotype of microglial cells to a more physiological-like one and, thereby, dampening the neurotoxic inflammatory response in the affected areas. Moreover, we demonstrated for the first time that the intracerebral infusion of αSynOs induced a systemic inflammatory response, characterized by a dysregulated cytokine/chemokine content in the serum, and associated with a peripheral cell infiltration within the SNC. Notably, pomalidomide reversed such a systemic pathological trait close to normality.

Immunomodulatory compounds are of increasing interest as a therapeutic strategy to target the neuroinflammatory component of PD, with the aim of slowing the progression of the neurodegenerative processes [5]. Several FDA-approved neuromodulatory compounds, including fingolimod (Gilenya), tacrolimus (Fujimycin), cyclosporin, and rapamycin, have shown neuroprotective properties in rodent models of dopaminergic degeneration [44–48]. As a result, rapamycin has been prioritized as a therapeutic candidate to move into clinical trials for repurposing in PD, based on solid preclinical evidence and its well-known neuromodulatory activity [49]. In contrast, IMiDs remain relatively poorly investigated in preclinical models of PD. Widely used and effective in cancer treatment, the relative dearth of preclinical research in PD models precludes their current proposition for clinical testing in this disorder. However, the latest generations of ImiDs offer several advantageous pharmacokinetic features. Notably, ImiDs are small molecular weight molecules that are orally bioavailable and have a greater brain uptake compared with classical immunosuppressants (brain/plasma concentration ratio rapamycin < 0.1) [50] making them potentially more suitable for treating chronic neurological disorders [1].

Supporting repositioning of IMiDs in neurological disorders in general, several studies have reported the beneficial effects of thalidomide or thalidomide-derivatives in Alzheimer disease (AD) preclinical models [51–54]. These studies have led to the clinical assessment of thalidomide in a cohort of mild to moderate AD patients [55], and of the derivative lenalidomide in a clinical trial aimed at evaluating potential beneficial effects in patients with mild cognitive impairment (MCI) and AD [56]. As regards to PD, the protective potential of thalidomide was first reported in MPTP-intoxicated mice [57], and both thalidomide and lenalidomide were subsequently evaluated in α-synuclein overexpressing mice, providing an improvement in both motor performance and neuropathological parameters [17]. Regrettably, despite preliminary promising results for the repurposing of IMiDs in PD, thalidomide and to a lesser extent lenalidomide, possess caveats that dampen enthusiasm for clinical translation in neurological disorders. Specifically, long-term use of both thalidomide and lenalidomide at doses required to mitigate inflammation has been associated with peripheral neuropathy and their well-known teratogenic effects [25, 28, 58–60]. Indeed, the poor tolerability of thalidomide hampered escalation to the desired dose purported efficacious in a recent AD clinical trial, resulting in trial failure [55]. Moreover, lenalidomide, although more potent than thalidomide in inducing anti-inflammatory effects, is a reported substrate for the p-glycoprotein efflux pump at the BBB, which may limit its CNS penetrance and maintenance of CNS therapeutic levels [61].

Pomalidomide, a third-generation analogue of thalidomide, possesses favorable physicochemical characteristics to support its potential CNS use by being in line with the Lipinski rule of 5, which predicts the successful delivery of a drug to the drug target after oral administration in physiological conditions [18]. In addition, pomalidomide possesses a promising CNS MPO (multiparameter optimization) score of 4.8 [1] that is predictive for an agent having desirable drug-like properties for neurological action. In previous studies, pomalidomide’s administration intraperitoneally to rodents as a suspension resulted in a brain/plasma concentration ratio in the range of 0.39 to 0.71 [27, 62, 63], thereby positioning this drug in the first line for repurposing in neurological disorders. Here, the pomalidomide bioavailability was further improved by the administration of the drug as a nanosuspension, which increases both the brain/plasma ratio and the brain concentration of the drug (Cardia et al. submitted) and allows low doses more readily to provide an effective brain concentration. Moreover, pomalidomide is far more potent than thalidomide or lenalidomide toward TNF-α inhibitory action [25, 26], which allows a drug dosing less likely to generate teratogenic and neurotoxic adverse actions but with potent anti-inflammatory activity [25, 28]. In the light of these features, pomalidomide is currently a first-choice drug for the treatment of select forms of tumors, with a dosing regimen 50-fold lower than thalidomide [64]. Up to the present study, pomalidomide has not been evaluated in mammalian models of PD. In a recent study, we reported its efficacy in a *Drosophila* LRRK2^WD40^ genetic PD model [16, 65]. In that study, we showed that LRRK2^WD40^ flies develop motor impairment and gradual loss of dopaminergic neurons with age [16], and pomalidomide administration through the diet prevented both age-dependent motor impairment and neuronal loss [16]. Supporting the neuroprotective property of this drug, pomalidomide was effective in reducing the ischemic brain injury in both mice and rats [27]. Moreover, both pomalidomide and its new derivative 3,6’-dithiopomalidomide (3,6’-DP) reduced cell loss in primary dopaminergic neuron cultures exposed to αSynOs [19].

In the present study, the neuroprotective activity of pomalidomide was characterized for the first time in a mammalian neuropathological model of PD, taking advantage of a recently developed model obtained by the intranigral infusion of toxic H-αSynOs in the rat [29]. Here, αSynOs were infused bilaterally within the SNpc in order to best model the clinical motor symptoms of PD. α-Syn-based models of PD reproduce critical pathological traits of the disease, epitomized by the α-Syn-induced progressive degeneration of dopamine neurons, spreading of aggregated α-Syn and a persistent inflammatory reaction in pathologically affected brain areas. These features position these models as highly applicable to study PD neuropathology and to test disease-modifying molecules targeting pathological mechanisms relevant to the model [31]. Specifically, small α-Syn aggregates that include oligomers, prefibrils and protofibrils are retained and, due to their degree of solubility, provide highly toxic species [66, 67]. Indeed, such soluble aggregates have been reported in degenerating areas within the brain and in biological fluids of PD patients [67–71]. In vitro and in vivo studies have suggested that oligomeric species induce the intracellular α-Syn aggregation and, in turn, promote the spreading of protein aggregates [31, 72–76]. Furthermore, αSynOs released from neurons establish contact with glial cells and stimulate pro-inflammatory responses in microglia [29, 77–81]. The small H-αSynOs used in the present study offer an additional advantage of being highly homogeneous in size and structure, being subjected to a standardized protocol to generate highly pure oligomers with defined structural properties [30]. This feature confers a high degree of reproducibility both within and across experiments in terms of the extent of neurodegeneration, as previously shown [29], which is pivotal when evaluating and comparing neuroprotective strategies.

Assessing neuroprotection in the α-Syn-based model used in the present study is, therefore, a crucial step to test molecules targeting the immune system in PD, as a prelude to clinical translation.

In line with our previous study [29], rats displayed significant motor deficits 3 months after the infusion surgery. Herein, we used three specific tests to probe different aspects of motor symptomatology, including sensorimotor deficits that were assessed via the beam challenging test and the gate test, and coordination impairment in fine movements execution that was evaluated via the vermicelli handling test [38, 39]. These tests support reliable measurement of typical impairments similarly evident in human PD identified as gait disturbances, such as stepping falls and bradykinesia [82]. Specifically, the beam challenging test is a validated behavioral task highly sensitive for motor deficits associated with partial degeneration of the nigrostriatal pathway, which has been previously used to test rodents in PD models [35, 36, 83]. The infusion of αSynOs induced an increase in the number of errors committed in the beam challenge test, which was reversed by pomalidomide. In the gait test, oligomer-infused rats exhibited a decrease in cadence (steps/s) and an increase in the time spent to traverse a fixed distance, expressed as a decreased velocity. Moreover, oligomer-infused rats displayed irregular, shorter strides that reflect the shortening in step amplitude in PD. Notably, all these motor impairments were mitigated by pomalidomide. Finally, the vermicelli handling test revealed an impairment in fine movements, which was again reversed by pomalidomide. In entirety, the results strongly forecast a beneficial action for pomalidomide in PD motor symptomatology.

Importantly, the efficacy of pomalidomide was underpinned by the rescue of dopaminergic cell loss, as revealed by the stereological count of nigral neurons. This analysis confirmed a significant cell loss in the αSyn-infused SNpc and the neuroprotective action of pomalidomide. The associated stereological count of Nissl-stained cells is of relevance as it confirms that changes in TH staining reflected changes in neuron number. It should be noted that pomalidomide treatment was initiated 1 month after H-αSynOs infusion, a time-point that may be considered to model the prodromal phase of PD, when mitochondrial damage and neuroinflammation are not yet associated with neuronal loss [29]. Therefore, the results of the present study suggest that pomalidomide can mitigate already initiated and ongoing neurodegenerative processes. This is pivotal in view of the translational relevance of this study, which suggests a potential disease-modifying effect of the drug treatment if given to early-diagnosed PD patients.

IMiDs are immunomodulatory compounds with potent anti-inflammatory properties, which were manifested in the present study by a dampening of the inflammatory response both within the brain and in peripheral blood. Intracerebral αSynOs infusion induced a chronic microglial reaction, revealed by an increase in Iba-1 IR, as previously shown in this as well as in other’s PD models, and in accord with the persistent microgliosis reported in the brain of PD patients [29, 83–88]. Moreover, reactive microglia displayed a pathological gain of toxic functions suggested by the increased production of the proinflammatory cytokine TNF-α, in line with an altered cytokine production, and elevations in reported levels in the cerebrospinal fluid of PD patients [89–91]. Notably, whereas in control rats the majority of microglia displayed low physiological levels of TNF-α, in oligomer-infused rats, two microglia subpopulations were recognized, displaying low and high TNF-α content, respectively, suggesting a supraphysiological production of the cytokine. Importantly, pomalidomide treatment suppressed the high TNF-α expressing microglia population leaving unaltered the low TNF-α expressing population, thereby restoring physiological levels of the cytokine. Moreover, pomalidomide augmented the microglia production of the anti-inflammatory cytokine IL-10, as expected by the immunomodulatory action of this drug. In synopsis, pomalidomide provided an allostatic regulation of microglia phenotype to restore the physiological pro/anti-inflammatory balance.

Of interest and, to our knowledge, not previously described in studies of intracerebral α-synuclein oligomer infusion was the development of a long-term peripheral inflammatory condition, characterized by the increased serum content of inflammatory cytokines and the concomitant decrease of anti-inflammatory mediators [92, 93]. This result is in line with an increasing literature reporting impaired serum cytokines in PD patients, and with the concept of PD as a systemic rather than CNS-specific disease, further validating our model as a translational model of PD [15]. Although the origin of systemic inflammation in PD remains uncertain, our results suggest that it may originate from the presence of toxic α-synuclein oligomers within the CNS, leaving an open question as to the sequence of mechanistic events.

Although there is a convergence of opinion on serum cytokine dysregulation in PD, conflicting findings have been reported with respect to single cytokines [94, 95]. Nonetheless, the elevated serum level of IL-17 present in our intracerebrally α-synuclein-infused rats is worthy of note, and is in line with the pivotal role currently attributed to this cytokine in neurodegenerative diseases and in glial cell activation [96]. Moreover, in line with a prevalence of the inflammatory immunophenotype in PD, IL-6 was significantly elevated, whereas IL-10 was significantly decreased in our model [15, 92, 93]. Finally, chemokines CXCL1/2 and RANTES were increased, in line with infiltration of immune cells into the inflammatory site, as revealed by CD3 immunostaining in αSynOs-infused rats [14, 97–99]. Interestingly, serum TNF-α was unchanged following intracerebral administration of αSynOs. Similarly, in a previous study, we found unchanged levels of serum TNF-α after dopamine depletion and after a chronic L-DOPA treatment, despite an intense inflammatory response within the brain [41]. Importantly, pomalidomide dampened the systemic inflammation, largely restoring normal cytokine levels within the serum. Pomalidomide was therefore able to effectively target the inflammation present in this PD model, resetting both the central and peripheral components to physiological, rather than pathological levels, and mitigating dopaminergic neuron losses and motor impairments.

Altogether, the present study provides compelling evidence of pomalidomide’s disease-modifying potential in a translational rodent model of PD to aid bridge the gap between preclinical and clinical studies, and fosters the repurposing of this drug for clinical testing in PD patients.

## Supplementary Information

Below is the link to the electronic supplementary material.

Supplementary file1 (PDF 963 kb)

Supplementary file2 (PDF 960 kb)

Supplementary file3 (PDF 982 kb)

Supplementary file4 (PDF 1021 kb)

Supplementary file5 (PDF 129 kb)

Supplementary file6 (PDF 528 kb)

Supplementary file7 (PDF 977 kb)

Supplementary file8 (PDF 163 kb)

Supplementary file9 (PDF 1064 kb)

Supplementary file10 (PDF 127 kb)

Supplementary file11 (PDF 859 kb)

Supplementary file12 (PDF 150 kb)

Supplementary file13 (PDF 130 kb)

Supplementary file14 (PDF 130 kb)

Supplementary file15 (PDF 710 kb)

Supplementary file16 (PDF 146 kb)

Supplementary file17 (PDF 145 kb)

Supplementary file18 (PDF 133 kb)

Supplementary file19 (PDF 131 kb)
